# Connecting the innate and adaptive immune responses in mouse choroidal neovascularization via the anaphylatoxin C5a and γδT-cells

**DOI:** 10.1038/srep23794

**Published:** 2016-03-31

**Authors:** Beth Coughlin, Gloriane Schnabolk, Kusumam Joseph, Himanshu Raikwar, Kannan Kunchithapautham, Krista Johnson, Kristi Moore, Yi Wang, Bärbel Rohrer

**Affiliations:** 1 Department of Ophthalmology, Medical University of South Carolina, Charleston, SC 29425, USA; 2 Research Service, Ralph H. Johnson VA Medical Center, Charleston, SC 29401, USA; 3 Alexion Pharmaceuticals, 352 Knotter Drive, Cheshire CT 06410, USA.

## Abstract

Neovascular age-related macular degeneration (AMD) is characterized by choroidal neovascularization (CNV). An overactive complement system is associated with AMD pathogenesis, and serum pro-inflammatory cytokines, including IL-17, are elevated in AMD patients. IL-17 is produced by complement C5a-receptor-expressing T-cells. In murine CNV, infiltrating γδT- rather than Th17-cells produce the IL-17 measurable in lesioned eyes. Here we asked whether C5a generated locally in response to CNV recruits IL-17-producing T-cells to the eye. CNV lesions were generated using laser photocoagulation and quantified by imaging; T-lymphocytes were characterized by QRT-PCR. CNV resulted in an increase in splenic IL-17-producing γδT- and Th17-cells; yet in the CNV eye, only elevated levels of γδT-cells were observed. Systemic administration of anti-C5- or anti-C5a-blocking antibodies blunted the CNV-induced production of splenic Th17- and γδT-cells, reduced CNV size and eliminated ocular γδT-cell infiltration. In ARPE-19 cell monolayers, IL-17 triggered a pro-inflammatory state; and splenocyte proliferation was elevated in response to ocular proteins. Thus, we demonstrated that CNV lesions trigger a systemic immune response, augmenting local ocular inflammation via the infiltration of IL-17-producing γδT-cells, which are presumably recruited to the eye in a C5a-dependent manner. Understanding the complexity of complement-mediated pathological mechanisms will aid in the development of an AMD treatment.

Age-related macular degeneration (AMD) is the leading cause of blindness in industrialized nations. The disease is found most commonly in adults age 50 or older, with an estimated, 1.75 million Americans currently diagnosed with advanced AMD. AMD gradually leads to the degeneration of the macula, the site of central, fine- tuned vision in the human eye. Advanced AMD occurs in two forms, dry (atrophic) and wet AMD^1^. Atrophic AMD is characterized by thinning or loss of the macular retinal pigment epithelium (RPE) and thickening of Bruch’s membrane (BrM), leading to atrophic region (geographic atrophy, GA). The appearance of increasing number of large drusen (crystalline deposits of extracellular material) as well as deposits (basal laminar and basal linear deposits) between the RPE and BrM are indicative of dry AMD. These deposits interfere with the hydraulic conductivity of BrM and impair the integrity of the RPE, which ultimately affects the health of the photoreceptors, resulting in retinal degeneration. A hallmark of wet AMD is choroidal neovascularization (CNV). In CNV, newly formed choroidal blood vessels grow through the RPE/BrM. Since new blood vessels more leaky, fluid will accumulate between the RPE and the retina, disrupting the connection between the photoreceptors and the RPE. Unless the fluid is drained and the retina allowed to reattach the photoreceptors will be lost, leading to loss of vision.

The development of AMD depends on a complex interplay of risk factors, which include age, genetics, and behavior^2^. Behavioral factors such as smoking^3^, diet^4^, and sunlight exposure^5^,^6^,^7^ each can contribute to the development of AMD; and genetic variations in genes involved in the complement system, as well as others have been found to be associated with risk for disease or risk of progression from early to late AMD^8^,^9^. Overall, the data suggest that AMD is a progressive neurodegenerative disease involving inflammation^10^, and in particular an inflammatory immune response^11^.

The immune system is divided into two distinct types - innate and adaptive. The innate immune system, which evolutionary, is much older than the adaptive immune system, consists of the complement system as well as different immune cell types that include phagocytes, mast cells, eosinophils, and basophils. The adaptive immune system, in which pathogenic exposure confers long-term defense memory in the host organism includes T- and B-lymphocytes. While both systems primarily protect the organism against invading pathogens, under disease conditions, self-cells can become targets for destruction and invading immune cells can cause damage to the host they are intended to protect. Finally, a number of different links exist that connect the innate and adaptive immune responses^12^, including the complement system^13^, and involving cell types that have functional characteristics of both systems, which includes B1-cells and γδT-cells^12^.

The complement system is initiated through three separate and independent pathways, the classical, the lectin, and the alternative pathway. These three pathways converge at the formation of a C3 convertase, C4bC2a (classical and lectin pathway C3 convertase) and C3bBb (alternative pathway C3 convertase), which then triggers activation of the common terminal pathway. As part of the terminal pathway, C3 and C5 convertase activation results in the production of the soluble anaphylatoxins C3a and C5a, which play a major role in mediating chemotaxis, inflammation, and the generation of cytotoxic oxygen radicals.

Anaphylatoxin receptors are G-protein coupled cell surface receptors expressed by many different cells. They have been demonstrated to be present on retinal pigment epithelium (RPE)^14^ and choroidal endothelial cells^15^ based on functional assays and receptor-mediated movement of leukocytes in the direction of the increasing concentration of anaphylatoxins has been demonstrated^16^.

Leukocytes fall into two categories, myeloid cells (neutrophils, monocytes, eosinophils and basophils) and lymphocytes (T-cells, B-cells and natural killer cells). In animal models of AMD a number of cells have been identified to infiltrate the eyes, including neutrophils and macrophages, natural killer cells and T-cells^17^; likewise, T-cells, macrophages and monocytes as well as other immune cells have been identified in eyes from AMD patients^18^,^19^. T-cells consist of four categories, T-helper cells (which includes Th1-, Th2- and Th17-cells), cytotoxic T-cells, γδT-cells, and T-regulatory cells. Importantly, the signature cytokine of Th17- and γδT-cells, IL-17 is significantly increased in human eyes with AMD^20^, and blocking IL-17 in eyes of mice with focal retinal degeneration was found to be neuroprotective^21^. Therefore, it was postulated that one or both of these cell types, Th17- and γδT-cells, contribute to inflammation and angiogenesis in the eye through the production of the IL-17 cytokine. Two recent publications suggest that the IL-17 present in the eye during age-dependent degeneration^22^ as well as in mouse choroidal neovascularization (CNV)^23^ is generated by γδT- rather than Th17-cells. Importantly, Hasegawa and colleagues showed by immunohistochemistry, flowcytometry, as well as quantitative QRT-PCR that γδT-cells invade the CNV-lesioned eye, accumulating in the CNV lesion. In addition, rag2-deficient mice that do not produce B- and T-cells can be reconstituted with γδT-cells but not conventional CD4^+^ T-cells in order to restore CNV sizes to wildtype levels^23^. Finally, in other systems such as chondrocytes and skin, IL-17 has been shown to increase VEGF production^24^ and VEGF promotes IL-17-producing γδT-cell accumulation^25^.

Based on the aforementioned data, we suggest that in the process of AMD disease pathogenesis, inadequate control of complement-driven inflammation results in the generation of the anaphylatoxin C5a, which is recruiting IL-17 producing γδT-cells to the eye. The IL-17 produced and released in the eye tissue might lead to an increase in VEGF-production, augmenting CNV.

## Results

### IL-17 expression in the CNV eye is correlated with the presence of a γδT-cell marker

Induction of severe CNV (40–50 burns per eye) was shown previously by Tsutsumi-Miyahara and colleagues to result in a transient increase in ocular infiltrating inflammatory cells measurable by flow cytometry in pooled eye samples. A small number of T-cells was found to be present between 12 hours and 7 days after induction of the lesions^17^. This observation was extended by Hasegawa and colleagues^23^ who demonstrated by immunohistochemistry, flowcytometry and quantitative QRT-PCR that γδT- rather than Th-17 cells are part of the invading T-cells, producing IL-17. Hence, here we relied on the presence of marker genes unique to γδT-cells (γδT-receptor), comparing CNV eyes with four carefully placed lesions to non-lesioned age-matched controls.

**Figure 1:**
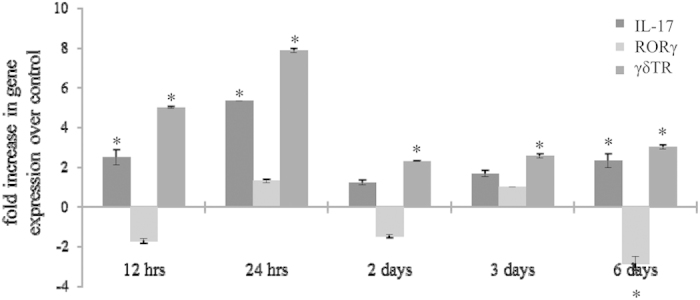
T-cell marker gene expression in the eye over time. Expression of IL-17, RORγ, and γδTR following CNV were measured at 12 hours, 24 hours, 2 days, 3 days, and 6 days. Levels of IL-17 mRNA peaked at 24 hours following CNV and remained elevated throughout 6 days. γδTR levels were similarly elevated through day 6 with a peak observed at 24 hours. RORγ levels remained unaltered in the presence of CNV. Data shown are average values (±SEM) per sample (n = 3). Significance required both a ±2-fold difference and *P *< 0.05 (indicated by*) between CNV and control samples.

To examine and confirm the presence and type of T-cells in eyes with CNV, RPE-choroid samples were analyzed. In preliminary data, we confirmed that T-cells are present 6 days after CNV induction, by showing that mRNA expression of CD3a (mature T-cells) and CD4 (T-regulatory cells and T-helper cells) was elevated in lasered eyes when compared to controls (fold change; CD3a: 8.0 ± 1.4; CD4: 9.1 ± 1.8). IL-17-producing T-cells, Th17- and γδT-cells both express the transcription factor RAR-related orphan receptor gamma (RORγ), but can be distinguished based on the presence of the γδT-cell receptor (γδTR) (Fig. 1). The levels of IL-17 mRNA peaked at 24 hours post CNV induction, and was continuously elevated by 2–3-fold up to 6 days (latest time point measured). Increased levels of IL-17 correlated with those of the γδT-cell receptor in the RPE-choroid fraction, whereas levels for RORγ were unaltered by the lesions.

The spleen contains, among other cells, B- and T-cells, which are exposed to antigens directly by filtering them from the blood, or indirectly by delivery by migratory macrophages or dendritic cells. Upon antigen presentation, T-cells can become activated, leading to clonal expansion. Six days after induction of CNV lesions, an equal increase in IL-17, RORγ and γδTR was measured in the spleen, suggestive of an overall activation of T-cells in the spleen (IL-17: 4.04 ± 0.39; RORγ: 4.96 ± 1.16; γδTR: 4.68 ± 0.34) (see control for Effects of C5a and C5 on T-cell marker gene expression).

**Figure 2:**
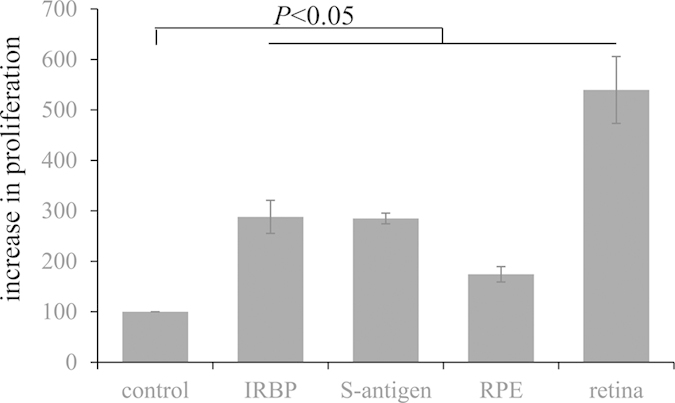
Splenocyte proliferation. Splenocytes of CNV animals were stimulated by various ocular antigens and cell proliferation was measured. Cells from naïve animals were used as controls. Splenocytes stimulated by the RPE/choroid (RPE) extracts as well as the retina proteins, IRBP and S-antigen, demonstrated a moderate increase (2–3-fold) in proliferation when compared to control; whereas stimulation with retinal extracts resulted in a much larger (6-fold) increase in cell proliferation. Data shown are average values (±SEM) per sample (n = 4–10).

If CNV lesions lead to an increase in T-cell activation, an overall T-cell immune responsiveness in primed T-cells (i.e., T-cells from CNV animals) should be measureable in response to antigenic stimuli from the eye. Spleens of CNV animals were collected and the resulting splenocytes stimulated *ex vivo* by the addition of antigenic stimuli (Fig. 2). General stimulation to ocular antigens was provided using retina and RPE-choroid extracts, whereas specific antigen stimulation was provided using IRBP and S-antigen, two well-known antigenic proteins that cause experimental autoimmune uveitis (EAU) in animals. IRBP is a glycoprotein in the interphotoreceptor matrix, S-antigen a soluble photoreceptor cell protein. Both proteins and/or other soluble retina- and RPE-derived proteins might gain access to the blood stream upon generating CNV lesions that break that blood retina barrier. In cells derived from CNV animals, exposure to RPE-cell extract caused a modest increase in cell proliferation, whereas retina-extract triggered a massive increase. Purified retina proteins (IRBP and S-antigen) did not mimic the large increase in proliferation seen in the retina-extract group, with both causing a significant but modest increase.

Taken together, our data thus far suggest that the generation of the CNV lesions, a mechanism that compromises the blood retina barrier, triggers an immune response that involves the adaptive immune response, leading to splenocyte proliferation and T-cell activation. Despite an overall increase in both Th17- and γδT-cell markers in the spleen and hence presumably an increase in both Th17- and γδT-cells in the blood stream, only a selective migration of γδT-cells into the eye appears to be associated with CNV.

### IL-17 expression in the CNV eye is reduced by blocking C5a production or signaling

How are γδT-cells recruited to the eye? T-cells have been shown to express C5a receptors (C5aR) on their surface, which could allow them to migrate toward a source of C5a present in the eye after the induction of CNV lesions^26^. C5a production and C5a-receptor signaling can be reduced by either inhibiting complement activation upstream of the C5 convertase, or by using blocking antibodies or antagonists to C5, C5a or C5aR, respectively. Here we took advantage of a blocking antibody against C5 (mouse IgG1), which has been used successfully to block C5-dependent antiphospholipid antibody-mediated thrombophilia^27^, as well as a novel antibody against mouse C5a (mouse IgG1). Mouse IgG1 antibodies were used since they have little or no antibody-dependent cellular cytotoxicity and complement-dependent cytotoxicity.

**Figure 3:**
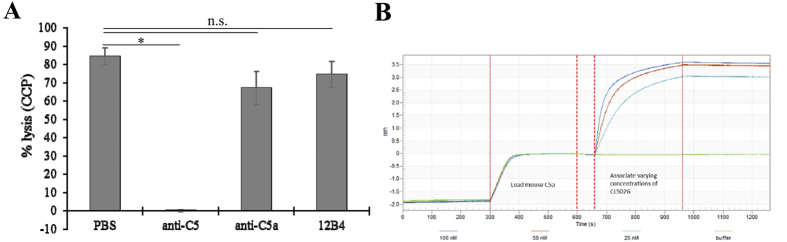
Characterization of antibodies. **(A)** Serum from mice injected with PBS, anti-C5, anti-C5a, and the antibody control 12B4 were analyzed for complement activation through use of a hemolysis assay. Serum from anti-C5 antibody treated animals was unable to lyse sheep red blood cells indicating successful blockage of complement activation. No significant difference was reported between lysis in mice injected with anti-C5a, PBS or 12B4. Data shown are average values (±SEM) per sample (n = 4–5). **(B)** Specificity of the monoclonal antibody specific for murine C5a was confirmed to bind to its target, murine C5a, with single digit nM affinity, using bio-layer interferometry.

To confirm efficacy of the anti-C5 blocking antibody, mice were injected with anti-C5 or control antibody and blood collected for hemolysis assays. This tests the functional capability of serum complement components of the classical pathway to lyse sheep red blood cells in a membrane-attack complex-dependent manner. Serum of mice injected with the anti-C5 antibody were unable to lyse sheep red blood cells, confirming successful blockage of complement activation, whereas lysis did occur in mice injected with the control antibody or the antibody against C5a (Fig. 3A). The monoclonal antibody specific for murine C5a was confirmed to bind to its target, murine C5a, with single digit nM affinity, using bio-layer interferometry. 10 μg/mL of biotinylated recombinant mouse C5a was loaded on streptavidin biosensors, and anti-C5a antibody (CLS026) associated at concentrations ranging from 25–100 nM, and then dissociated. Local fits using the Octet analysis software calculate a KD of 8 nM (Fig. 3B).

**Figure 4:**
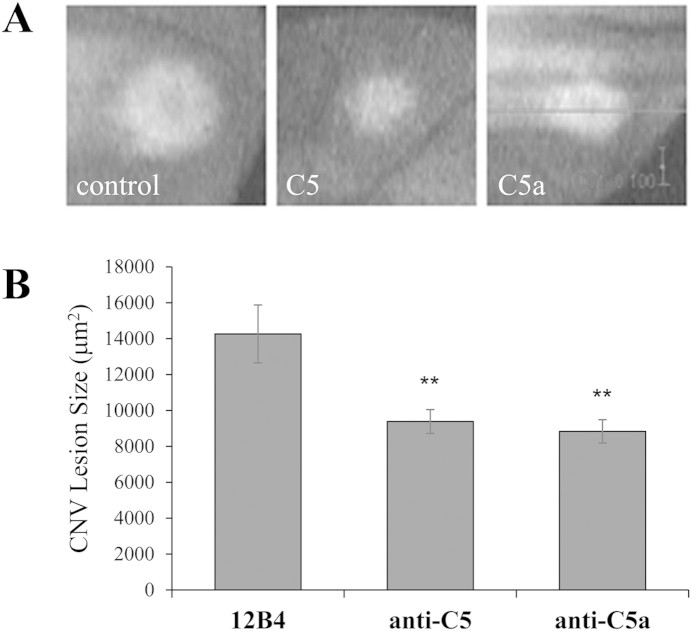
CNV is reduced in animals injected with anti-C5 and anti-C5a. Following laser-induced CNV, OCT was used to measure lesion size in the presence of anti-C5, anti-C5a, or 12B4 (control). OCT images show a decrease in lesion size with treatment of anti-C5 and anti-C5a when compared to control **(A)**. Quantification of these results **(B)** indicated a nearly 40% decrease in lesion size when injected with anti-C5 and anti-C5a (*P ≤ 0.01). Data shown are average values (±SEM) per lesion (n = 7 animals per condition). Scale bar: 100 pixels or 160 μm.

After having confirmed that the antibodies can be used as blocking antibodies and/or bind to their targets, the antibodies were tested in the mouse CNV model. The development of CNV following laser photocoagulation was assessed in 4 cohorts of mice (mice injected every 48 hours intravenously with PBS, control antibody, anti-C5 or anti-C5a) at 3-months-of-age. On day 5 after CNV induction, CNV size was measured using OCT (Fig. 4A); 6 days after CNV induction, mice were sacrificed and tissues collected. Previous reports have shown that CNV development is significantly reduced in mice in C5^−/−^28 or C5a-R^−/−^ mice^29^ or mice injected intraocularly with rat IgG2a C5a blocking antibodies^29^. Here we confirmed and expanded these results demonstrating that CNV development was significantly reduced in mice treated with mouse IgG1 C5- (9,384 ± 665 μm^2^) or C5a-blocking (8,839 ± 650 μm^2^) antibodies when compared to control antibody-injected mice (14,260 ± 1614 μm^2^; *P* ≤ 0.01; Fig. 4B). Both treatments reduced CNV by ~40% when compared to the controls.

**Figure 5:**
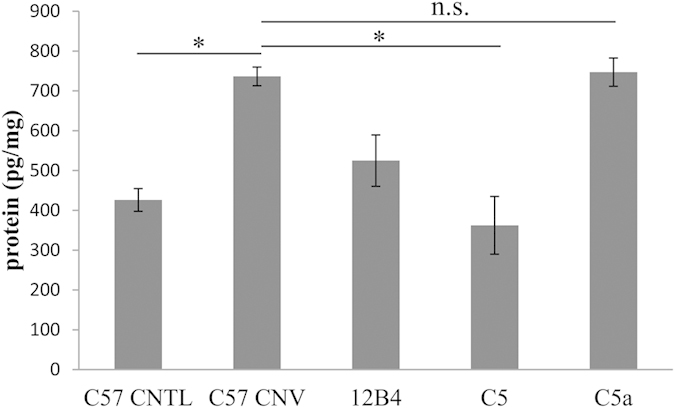
Animals injected with anti-C5 have lower ocular anti-C5a levels. ELISA measurements of RPE/choroid demonstrated an increase of C5a levels after induction of CNV (*P ≤ 0.001). This increase was eliminated in anti-C5-treated mice; whereas mice treated with anti-C5a and 12B4 control antibodies had control levels of ocular C5a. Data shown are average values (±SEM) (n = 6–8 animals per condition).

ELISA measurements of RPE-choroid confirmed that CNV induction lead to an increase in levels of C5a^26^. Treatment with a C5 blocking antibody, which prevents the generation of C5a, resulted in the elimination of the CNV-induced increase in C5a levels, while animals treated with the C5a-blocking antibody, retained elevated C5a levels; but C5a is presumably bound to the antibody and thereby inactivated (Fig. 5).

**Figure 6:**
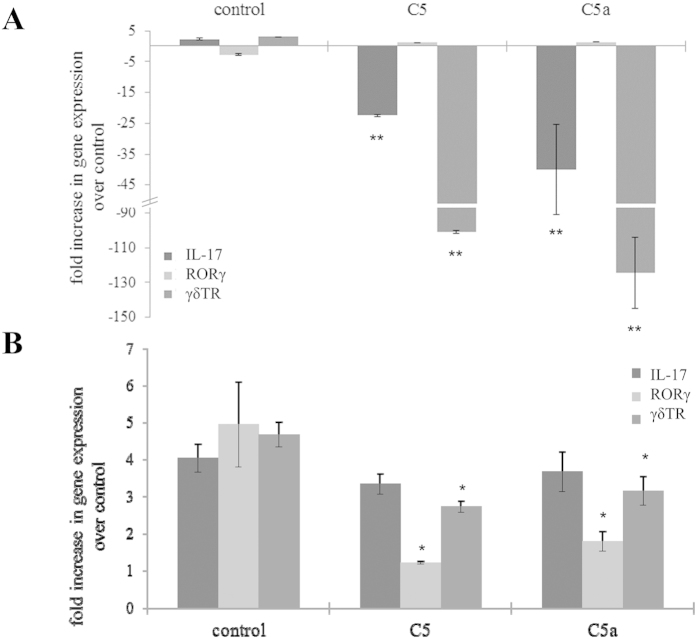
Effects of C5a and C5 on T-cell marker gene expression. Splenic **(B)** and ocular **(A)** samples were isolated 6 days after induction of CNV and analyzed by QRT-PCR using primers specific for Th-17 (RORγ) and γδT-cells (γδTR). **(A)** Following CNV, mice treated with anti-C5 and anti-C5a demonstrated a significant decrease in ocular levels of IL-17 and γδTR gene expression, whereas RORγ levels were unaltered. **(B)**. Splenic levels of T-cell-specific genes in CNV mice indicated that RORγ levels returned to control levels in mice treated with anti-C5 and anti-C5a, whereas γδTR remained significantly elevated (*P < 0.05). Data shown are average values (±SEM) (n = 3).

How does complement inhibitor treatment affect the activation of Th-17 and γδT-cells in the spleen and their recruitment to the eye? Both anti-C5 and anti-C5a had small but significant effects in reducing the CNV-triggered increase in splenic γδTR levels; whereas the CNV-triggered increase in RORγ levels was completely prevented in the treated mice (Fig. 6A). However, inhibitor treatment completely prevented the rise of IL-17 and γδTR in the eyes of CNV mice (Fig. 6B), supporting the hypothesis that C5a levels in the eye contribute to the recruitment of C5a-receptor-bearing T-cells.

### 3) IL-17 promotes inflammation in the eye

Hasegawa and colleagues have recently shown that depletion of γδT-cells reduced IL-17 levels in the eye and ameliorated experimental CNV^23^.

**Figure 7:**
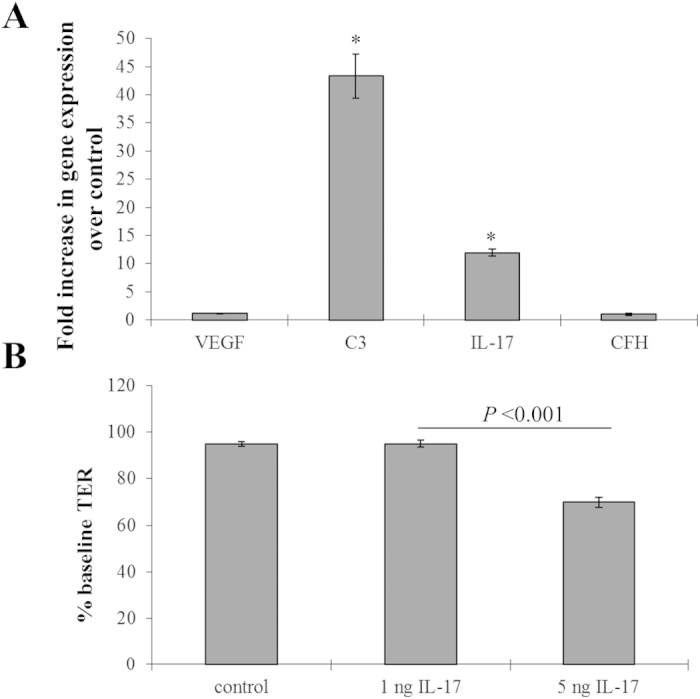
Effect of IL-17 on RPE cells. **(A)** Change in gene expression following apical IL-17 exposure (5 ng/mL) was measured in mature ARPE-19 cell monolayers. C3 as well as IL-17 expression levels demonstrated an increase in fold change over the control, whereas VEGF and CFH were unaltered. **(B)** Transepithelial resistance measurements indicated a loss in barrier function in response to apical application of 5 ng/mL IL-17 after four hours. Data shown are average values (±SEM) (n = 3).

Here we confirmed the pro-angiogenic effect of IL-17 in RPE cells by stimulating ARPE-19 cells grown as mature monolayers^30^ and measuring gene expression for marker genes and barrier function. ARPE-19 cells were chosen since when grown as monolayers they express all the signature genes of human RPE cells^31^, they develop tight junctions and resemble an aged RPE^32^. A greater than 40-fold increase in C3 gene expression as well as a ~10-fold increase in IL17 was observed following IL-17 stimulation, whereas expression levels of VEGF and CFH mRNA were unaffected (Fig. 7A). Addition of 5 ng of IL-17A into the apical chamber of the monolayer resulted in a significant decrease in transepithelial resistance as measured using a volt-ohm meter (Fig. 7B).

## Discussion

The main results of the current study are: 1) CNV triggered an immune response in the spleen presumably via the release of soluble retina or RPE proteins, resulting in an increase in IL-17-producing γδT- and Th17-cells based on marker gene expression. 2) Despite this increase in systemic γδT- and Th17-cells, there is only evidence for γδT-cell migration into the CNV eye. 3) A blocking antibody to C5 or reducing C5a-signaling reduced CNV in the mouse eye, blunted the CNV-induced production of Th17- and γδT-cells in the spleen, and prevented the rise of IL-17 and γδT-cell receptor mRNA in the CNV eyes. 4) Application of exogenous IL-17 triggered a pro-inflammatory state in RPE cells, resulting in an increase in IL-17 and C3 production. 5) Thus in summary, our data suggest that CNV lesions trigger a splenic immune response that augments ocular inflammation via the infiltration of IL-17-producing γδT-cells recruited to the eye by the locally generated chemoattractant C5a.

### AMD as an immune disease

The potential roles of both the innate and the adaptive immune system in AMD and its models are currently being investigated. Here, we were able to demonstrate an increase in T-cell marker gene expression (CD3a, CD4, RORγ and γδTR) in the spleen in response to CNV as well as an increase in splenocyte proliferation in response to retina- and RPE-derived antigens. First, this clearly speaks to the loss of the immune privilege of the retina in CNV, a pathology that involves the loss of the blood retina barrier. Second, while the antigenic molecules that activate γδT-cells are still largely unknown, it suggests that they might include ocular inflammation-associated proteins. Finally, while we have focused on the role of IL-17 in the context of the recruitment of γδT-cells, another aspect that requires attention is the fact that γδT-cells appear to respond to self-molecules that signal potential danger or cellular stress^33^, which places them between the rapid innate immune system and the slower adaptive immune system. In support of a role of the adaptive immune system, there are a number of reports documenting the presence of autoantibodies in AMD subjects. Antibodies (IgGs) are generated randomly or in response to a foreign substance. Those that recognize self-cells or self-proteins and non-threatening environmental compounds are eliminated through clonal deletion; yet some escape this screening mechanism to generate autoantibodies (aAbs) that attack “self”, leading to inflammation and damage. Recent studies have suggested that aAbs might play a role in AMD pathogenesis. AMD patients have elevated levels of IgG aAbs when compared to controls^34^, with ligands such as GFAP^35^, carboxyethylpyrrole (CEP)^36^, α-crystallin, α-enolase^35^, annexin II^37^ or cardiolipin^38^. Proof of concept that aAbs generate disease was provided by immunizing mice with CEP-adducted BSA, which lead to pathology similar to dry AMD. Importantly, immunizing rag1^−/−^ mice which are deficient in B- and T-cells, no anti-CEP antibody and no pathology was detected^39^. Natural antibodies (nAbs) on the other hand, are the most evolutionarily conserved antibodies and recognize specific antigens without prior exposure and immunization to these antigens^40^. They are IgM antibodies that are generated by B-1 cells. The host defense system utilizes these nAbs to quickly respond to common microbial pathogens (recognition of non-self);^41^ in addition, nAbs also contribute to tissue homeostasis by removing apoptotic cells, or by triggering complement activation on damaged membranes or pathological structures^42^. A screen utilizing antigen microarrays (~70 neoepitopes), revealed a small set of nAbs that correlated with dry or wet AMD diagnoses^43^. We have shown in the mouse model of CNV that neoepitopes for nAbs are present in CNV lesions and that rag1^−/−^ can be reconstituted with these specific nAbs for the augmentation of CNV size^44^. Hence, while the animal data support a role of the immune response in AMD, the involvement of aAbs or nAbs in AMD remains unclear. We will further investigate these questions in future studies.

### IL-17 in AMD

IL-17 is a major proinflammatory cytokine that is linked to the pathogenesis of a number of different diseases including rheumatoid arthritis, uveitis and possibly AMD. Relevant for the development of AMD, which for the wet form involves an increase in VEGF production and secretion as well as endothelial cell growth and vessel formation, IL-17 has been shown in other systems to not only increase production of VEGF^45^, but to induce angiogenesis, cell migration, and cell invasion using human dermal endothelial cells^46^. In animal models relevant to AMD, IL-17 has been found to accumulate in the mouse eye during age-dependent degeneration^22^ as well as during CNV^23^, and CNV progression can be reduced by interfering with IL-17 signaling^23^. Finally, in AMD patients, increased serum levels of IL-17 have been reported^47^ as well as hypomethylation of the IL-17 receptor C^48^. There are a number of different effector cells that produce IL-17; the IL-17-producing T-cell (Th17), γδT-cells, as well as innate lymphoid cells^49^. In the mouse models relevant to AMD, IL-17 in the eye is due to the infiltration of γδT-cells rather than Th17-cells^22^,^23^. However, the cellular origin of IL-17 in human AMD has not yet been investigated. Our data support the hypothesis that γδT- rather than Th17-cells are the T-cells producing IL-17 in the eye in response to CNV, since the increase in IL-17 observed in the eyes of control animals correlated with an increase of the γδT-cell receptor, rather than a general marker for T-cells (RORγ). Our data confirm and extend the data by Hasegawa and colleagues^23^ who demonstrated that infiltrating γδT- rather than Th17-cells are the main source of IL-17 in CNV eyes by providing a mechanism for recruitment. Likewise, our data support the observation that IL-17 generates a pro-inflammatory environment in the RPE by affecting barrier function as reported previously by Chen and colleagues^50^, increasing VEGF and complement production, overall generating a vicious cycle of inflammation and complement activation^51^.

### How to link complement and IL-17 - C5a as a chemoattractant

It is now accepted that an overactive complement system is tied to the incidence of AMD. There exists a high concentration of complement regulatory proteins and membrane attack complex (MAC) in the area of Bruch’s membrane and RPE^52^,^53^,^54^,^55^ and membrane attack complex deposition density is correlated with AMD risk genotypes^56^. The hypothesis that the alternative pathway of complement (AP) is critical to AMD pathogenesis was strengthened by reports showing that a polymorphism in the AP control protein factor H is strongly associated with AMD^57^,^58^,^59^,^60^. In addition, variations in the genes for CFB, C2, C3, CFHR1/3^61^,^62^,^63^ as risk factors have also been reported; and an inverse relationship between AMD and SERPING1 (C1 inhibitor) exists^64^. Finally, anaphylatoxin proteins C3a and C5a have been reported in pathological structures associated with AMD^29^. Of the biological effector molecules produced during complement activation, only the anaphylatoxins have been shown to exhibit proangiogenic and chemotactic properties. C5a has been shown to promote IL-22 and IL-17 expression from CD4^+^ T-cells derived from AMD patients^47^, and C5a has been shown to promote production of another cytokine, IL-8^65^, as well as VEGF^66^, by ARPE-19 cells. Regarding C5a’s chemotactic properties, while T-cells have been shown to express C5a receptors^26^, no data are available in the ocular space that supports the notion that T-cells indeed follow the C5a gradient to enter the eye in AMD or in models of AMD. Our data here suggest for the first time that the anaphylatoxin C5a that is generated in the eye in response to CNV^26^, and that is reduced in response to the C5 blocking antibody or by reducing complement activation^26^, could mediate the recruitment of pro-inflammatory T-cells into the eye. However, additive effects of removing direct effects of C5a on RPE or choroidal endothelial cells together with the lack of recruitment of γδT-cells cannot be excluded. Likewise, it cannot be excluded that in the presence of the antibodies, IL-17 producing γδT-cells might still migrate to the eye, but lose their differentiation status as has been shown by the loss of function of T-regulatory cells^67^, a question that however goes beyond the scope of this manuscript. Importantly, RPE cells have been shown to produce various cytokines in response to C5a stimulation^68^ and C5a was shown to interfere with anti-immunogenic role of the RPE by suppressing the production of the immunosuppressive agent TGFβ and decreasing the RPE’s ability to suppress immune cell proliferation^69^.

### Does this work point us toward a target in AMD?

Zarbin and Rosenfeld^51^ recently summarized the current concept of pathology and treatment strategies for AMD. Based on the available evidence, oxidative stress leads to the oxidative damage in the photoreceptors, RPE, Bruch’s membrane and choroid, in particular in the macula. This includes the accumulation of lipofuscin^70^ as well as the generation of neoepitopes^44^,^71^, triggering chronic inflammation involving both the innate and adaptive immune response, including the complement system. Chronic inflammation, in particular in the presence of complement and other genetic variations, will lead to structural damage in the ocular structures sensitized and marked by oxidative stress, leading to the accumulation of extracellular debris and changes in the extracellular matrix^72^. Together, these changes will eventually lead to drusen and pigmentary changes, geographic atrophy and/or choroidal neovascularization. Since oxidative stress will not only trigger inflammation and complement activation, but a powerful feedback loop exists between inflammation, complement activation and oxidative stress in the RPE^44^ and presumably in other ocular structures, a vicious cycle is present in AMD, advancing the disease. Based on this concept, targets for intervention have included antioxidants^73^, compounds that reduce the efficacy of the retinoid cycle^74^, anti-inflammatory agents^75^, compounds that target the complement system^76^, as well as anti-angiogenic compounds^77^. Here we have confirmed and extended results by Nozaki and colleagues that systemically rather than intraocularly administered blocking antibody that interferes with C5a production or signaling reduces CNV^29^ and extended the clinical data from the COMPLETE study^78^ that anti-C5 blocking antibody treatment at a concentration that completely blocks the common terminal pathway systemically, can reduce CNV. Importantly, here we used mouse IgG1 rather than rat IgG2a^29^ antibody; rat IgG2a, just like IgM, human IgG1, mouse IgG2a, rabbit IgG and other antibodies all fix and activate complement, whereas murine IgG1 does not. Additional experiments for this antibody are required to determine the optimal ocular concentration to determine efficacy in additional animal models. Likewise, we confirmed and extended data by Hasagawa and colleagues that depleting γδT-cells reduced IL-17 levels and ameliorated experimental CNV^23^. Specifically, here we showed that lack of recruitment of IL-17-expressing γδT-cells to the eye reduced CNV. With respect to the vicious cycle present in AMD, we added another component to this concept. Here we showed that complement activation via the production of the anaphylatoxin C5a leads to the recruitment of IL-17 producing T-cells. IL-17 as a chemokine was found to lead to the upregulation of IL-17 and C3 expression from RPE monolayers, increasing the angiogenic profile of the RPE. Interestingly, IL-17 did not alter VEGF expression. This suggest that IL-17 may function as a proangiogenic factor in a VEGF-independent manner, a hypothesis already posed by Hasegawa and colleagues and extended to suggest that “anti-IL-17 therapy could be useful for patients resistant to anti-VEGF therapy”^23^. Overall, our data seem to suggest that the reduction of complement activation upstream of and including C5a production would prevent chemotactic attraction of cytokine-producing lymphocytes, membrane attack complex activation and reduce the inflammatory- and oxidative stress-mediated feedback loop, possibly providing an efficient target to break the vicious cycle in the pathophysiology of AMD.

## Material and Methods

### Animals

C57BL/6J mice were generated from breeding pairs (Jackson Laboratories, Bar Harbor, ME). Animals were housed under a 12:12 hour, light:dark cycle with access to food and water ad libitum. All experiments were performed in accordance with the ARVO Statement for the Use of Animals in Ophthalmic and Vision Research and were approved by the Medical University of South Carolina Institutional Animal Care and Use Committee under the protocol number 2490.

### CNV lesions

CNV lesions were induced as described previously^79^. In short, 3- to 4-month-old mice were anesthetized (xylazine and ketamine, 20 and 80 mg/kg, respectively) and pupils dilated (2.5% phenylephrine HCl and 1% atropine sulfate), using argon laser photocoagulation (532 nm, 100 μm spot size, 0.1 s duration, 100 mW) to generate four laser spots per eye surrounding the optic nerve, using a handheld coverslip as a contact lens. Any laser spots not creating a lesion (indicated by bubble formation), or those accidentally rupturing a blood vessel were excluded from size determination by coherence (OCT) analysis.

### CNV lesion analysis

CNV size determination was accomplished by OCT analysis, using the SD-OCT Bioptigen® Spectral Domain Ophthalmic Imaging System (Bioptigen Inc., Durham, NC). Mice were anesthetized and eyes hydrated with normal saline. Using the Bioptigen® *InVivo* Vue software, rectangular volume scans were performed (1.6 × 1.6 mm; 100 B-scans, 1000 A-scans per B scan), and using the systems *en face* fundus reconstruction tool the center of the lesion was determined and the image saved. ImageJ software (Wayne Rasband, National Institutes of Health, Bethesda, MD: available at http://rsb.info.nih.gov/ij/index.html) was then used to measure the area around the hyporeflective spot produced in the fundus image^80^. Based on the size of the individual pixels (1.6 × 1.6 μm) the lesion sizes were calculated.

### Complement inhibitors

Two blocking antibodies were used. CLS026 is a mouse IgG1 antibody specific for a neoepitope on murine C5a. CLS026 was derived using a phage display selection on murine C5a, with negative selection against full length human C5. The C5 antibody (BB5.1) is a mouse IgG1 antibody raised against purified mouse C5^81^. BB5.1 recognizes both the disulphide linked alpha-chain (111 kDa) and beta-chain (75 kDa) of mouse C5 and inhibits C5-dependent hemolysis. A mouse IgG1 antibody (135.8) raised against human complement component C8 was used as an isotype control^82^. All three antibodies were each stably expressed in Chinese hamster ovary (CHO) cells and purified using single step affinity chromatography with mabselect Xtra (GE) protein A resin. Antibodies were free of endotoxin and determined to be greater than 95% pure using capillary electrophoresis.

### Complement hemolysis assay

Terminal complement activity in recipient mouse sera was determined by standard methods to assess its ability to lyse chicken erythrocytes, which had been presensitized with erythrocyte-specific Abs as previously described^83^. Briefly, purified anti-C5 mAb at 100, 2, and 0 μg/ml in gelatin Veronal-buffered saline (GVBS) containing 0.1% gelatin, 141 mM NaCl, 0.5 mM MgCl2, 0.15 mM CaCl2, and 1.8 mM sodium barbital was used as low, medium, and 100% lysis controls, respectively. Experimental samples were prepared by diluting the murine test serum 1/10 in GVBS. Control and experimental samples were added, in triplicate, to wells of a 96-well plate containing an equal volume of 10% normal Balb/c mouse serum and 10% human C5-deficient serum in GVBS. Two microliters of 500 mM EDTA was added to the third well of both the 100% lysis and experimental sample triplicates to generate “no hemolysis” color control standards for each condition^83^. Chicken erythrocytes were washed in GVBS, sensitized by incubation with an anti-chicken RBC polyclonal Ab (Intercell Technologies; 0.1% v/v) at 4 °C for 15 min, washed again, and resuspended in GVBS at a final concentration of ∼7.5 × 10^7^ cells/ml. The sensitized chicken erythrocytes (∼2.5 × 10^6^ cells) were added to the plate containing the controls and samples, mixed briefly on a plate shaker, and incubated at 37 °C for 30 min. The plate was then mixed again, centrifuged at 3000 rpm for 3 min, and 80 μl of the supernatant was transferred to wells of a 96-well flat-bottom microtiter plate (BD Biosciences). The plate was read at OD415 using a microplate reader and the percentage of hemolysis was determined using the following formula: % hemolysis = 100×((OD sample–OD sample color control)/(OD 100% lysis control–OD 100% lysis color control)).

### Bio-layer interferometry

Bio-layer Interferometry (BLI) was used to confirm that CLS026 bound to its target, mouse C5a. 10 μg/mL of biotinylated recombinant mouse C5a was loaded on streptavidin biosensors (Pall/Fortebio) and assays conducted according to the manufacturer’s recommendations. Following load, CLS026 in 1× kinetic buffer was associated with the biosensors at varying concentrations. All binding data were collected at 30 °C. The experiments included five steps: baseline (0–300 seconds); CLS026 loading onto sensors (300–600 seconds); second baseline 600–650 seconds); association of CLS026 for measurement of K_on_ (650–950 seconds); and dissociation of CLS026 for the measurement of K_off_ (950–1250 seconds). Baseline and dissociation steps were conducted in 1x kinetic buffer. Local fits using the Octet analysis software were used to calculate the KD.

### C5a ELISA

For the quantitative determination of mouse C5a in RPE/choroid tissue homogenates, a sandwich enzyme immunoassay was used according to the manufacturer’s instructions (Kamiya Biomedical Company; Seattle, WA). In short, pre-coated plates were exposed to the antigen for 2 hours at 37 °C, washed and incubated with detection antibody to C5a followed by peroxidase-conjugated secondary antibody and color development using TMB substrate. The concentration of C5a in the ocular samples were determined by comparing the O.D. of the samples to a calibration curve (calibrators provided in the kit).

### Quantitative RT-PCR (QRT-PCR)

To assess mRNA levels for genes of interest, ARPE-19 cells or RPE-choroid-sclera (referred to as RPE-choroid) fractions isolated from control and CNV eyes were utilized and processed as described before^14^,^79^. In short, real-time PCR analyses were performed in triplicate in a GeneAmp® 5700 Sequence Detection System (Applied Biosystems, Foster City, CA) using standard cycling conditions. Quantitative values were obtained by the cycle number. Significance required both a ±2-fold difference and *P* < 0.05 between the relevant comparisons. Primers used are listed in Table 1.

### ARPE-19 cells

ARPE-19 cells, a human RPE cell line that displays the differentiated phenotype of RPE cells, were grown as described previously^30^. Cells were expanded in DMEM-F12 (Gibco) with 10% fetal bovine serum (FBS) and 1× penicillin:streptomycin until they reach confluence. To promote formation of stable barrier facilities, serum was reduced to 2%. Barrier function was assessed based on transepithelial resistance (TER) measurements^30^. FBS was removed completely for two days, which does not alter survival or monolayer formation, such that cells can be treated with a known concentration of IL-17.

### Splenocyte proliferation

Cell proliferation assays were performed as published previously^84^. In short, cell suspensions of splenocytes were prepared and the concentration of cells adjusted to 5 × 10^6^ cells/mL. The cells were grown in RPMI-complete medium containing RPMI-1640 (Gibco BRL Carlsbad, CA), 10% FBS, 1x penicillin:streptomycin, 1 mM glutamine, 1 mM nonessential amino acids, and 500 μM 2-ME (Sigma-Aldrich; St. Louis, MO). Splenocytes were stimulated with IRBP161–180 (20 g/mL), S-antigen (concentration), and supernatants of solubilized RPE/choroid or retina extracts for 72 h. For proliferation at 48 hours, 1 Ci of [methyl-3H] thymidine (Amersham Biosciences Pittsburgh, PA) was added to each well of the plate and the mean incorporation of thymidine into DNA was measured at 72 hours by a 1450 Microbeta Wallac Trilux Liquid Scintillation Counter (Perkin-Elmer Life Sciences, Waltham, MA).

### Statistics

For data consisting of multiple groups, one-way ANOVA followed by Fisher’s post hoc test (*P* < 0.05) was used; single comparisons were analyzed by Student *t* test analysis (*P* < 0.05); normalized data were analyzed using a *Z*-test (*P* < 0.05).

## Additional Information

**How to cite this article**: Coughlin, B. *et al.* Connecting the innate and adaptive immune responses in mouse choroidal neovascularization via the anaphylatoxin C5a and γδT-cells. *Sci. Rep.* **6**, 23794; doi: 10.1038/srep23794 (2016).

## Figures and Tables

**Table 1:** Mouse and human primer sequences for QRT-PCR analysis.

Mouse Primers	Forward	Reverse
β-actin	5′-AAATCTGGCACCACACCTTC-3′	5′-GGGGTGTTGAAGGTCTCAAA-3′
C3	5′-TCAGATAAGGAGGGGCACAA-3′	5′-ATGAAGAGGTACCCACTCTGGA-3′
C5	5′-CAGGGTACTTTGCCTGCTGA-3′	5′-TGGATTTTCATGGTGGGGCA-3′
Vegf	5′-CTGGACCCTGGCTTTACTGC-3′	5′-TGAACTTGATCACTTCATGGGACT-3′
IL-17	5′-TTCAGGGTCGAGAAGATGCT-3′	5′-AAACGTGGGGGTTTCTTAGG-3′
ROR gamma	5′-CGACTGGAGGACCTTCTACG-3′	5′-TTGGCAAACTCCACCACATA-3′
T-cell Receptor	5′-CAGGCACTTACATCCACTGGT-3′	5′-TGAATCTGGAATCCACCACAG-3′

Human Primers	Forward	Reverse
β-actin	5′-AAATCTGGCACCACACCTTC-3′	5′-GGGGTGTTGAAGGTCTCAAA-3′
C3	5′-ACCACACCCTCCAAACAAAG-3′	5′-ACTGTCTTCTCCACGGTGCT-3′
VEGF	5′-TCTTCAAGCCATCCTGTGTC-3′	5′-ATCCGCATAATCTGCATGGT-3′
IL-17	5′-GCAATGAGGACCCTGAGAGA-3′	5′-TGGATGGGGACAGAGTTCAT-3′
Factor H	5′-AGAAGGCACCCAGGCTATCT-3′	5′-CACAGGGCCTTTTCTGACAT-3′
